# Effect of Si on the Energy Band Gap Modulation and Performance of Silicon Indium Zinc Oxide Thin-Film Transistors

**DOI:** 10.1038/s41598-017-15331-7

**Published:** 2017-11-13

**Authors:** Jun Young Choi, Keun Heo, Kyung-Sang Cho, Sung Woo Hwang, JaeGwan Chung, Sangsig Kim, Byeong Hyeon Lee, Sang Yeol Lee

**Affiliations:** 1 Department of Electrical Engineering, Korea University, Seoul, 136-701 Republic of Korea; 2 Device Lab, Samsung Advanced Institute of Technology, Suwon-si, 443-803 Republic of Korea; 3 Platform Technology Lab, Samsung Advanced Institute of Technology, Suwon, 443-803 Republic of Korea; 4 Department of Semiconductor Engineering, Cheongju University, Cheongju, 360-764 Republic of Korea; 5 Research Institute of Advanced Semiconductor Convergence Technology, Cheongju, 360-764 Republic of Korea

## Abstract

The band gap properties of amorphous SiInZnO (a-SIZO) thin-film transistors (TFTs) with different Si concentrations have been studied. The electronic structures of the films, engineered by controlling the Si content, have been investigated through the changes of the band gap and band edge states. Carrier generation at oxygen vacancies can modify the band gap states of oxide thin films. Si suppresses the number of oxygen vacancies—which are carrier generation sites—so shifts the Fermi energy level away from the conduction band. It is difficult to derive the electronic structures of amorphous oxide semiconductors by electrical measurements. Thus, we used a combination of ultraviolet photoelectron spectroscopy, Kelvin probe measurements, and electron energy loss spectroscopy to measure the band gap and electrical performance variations of SIZO TFTs with Si doping. To verify the versatility of Si doping in modulating electronic properties, high-performance depletion-mode inverter circuits consisting of 0.1 to 0.3 wt% Si-doped a-SIZO TFTs were fabricated. These inverter models operate through the threshold voltage difference that arises from the different Si contents. High voltage gains of ~20.62 at a supply voltage of 15 V were obtained with the two TFTs, with a strong dependence on the subthreshold swing.

## Introduction

Amorphous oxide semiconductors (AOSs) have been extensively researched as promising channel materials for thin-film transistors (TFTs) applied in next-generation displays^1–3^. Among them, functionalised zinc oxide (ZnO) materials have attracted much attention as one promising candidate for high-performance flexible displays due to their excellent electrical characteristics and optical properties^4–6^. Therefore, extensive research has been conducted on the application of Zn-based AOS-TFTs. Especially, indium–zinc–oxide (IZO)-based systems are the leading active layer candidates for high-performance backplanes, as they can form semiconductor and electrode layers that offer unique electrical and mechanical advantages. The high processing temperatures of over 250 °C reached during the post-annealing and/or passivation processes are critical barriers to the use of flexible plastic substrates, such as polycarbonate, polyether sulfone, and polyethylene terephthalate that require processing temperatures below 150 °C.

In our previous reports, we investigated the good electrical performance of amorphous silicon–indium–zinc–oxide (a-SIZO) TFTs adapted to flexible devices, owing to their low processing temperatures of below 150 °C^7–9^. In this study, by taking advantage of the low processing temperature (<150 °C) and optimizing the processing steps, we achieved a-SIZO TFTs with higher mobilities and improved electrical characteristics as compared to those obtained in the previous studies. The origin of the defect state was involved with the creation of oxygen vacancies (V_O_)^10^. Carrier generation could modify the position of the Fermi level within the band gap of the oxide thin film. To design a high-performance device, the structure of the interface between the metal electrode and active thin-film channel layer must be optimised, so an exact analysis of the energy band gap of the thin film is necessary. However, because of the difficulties in deriving the relationship between the stability and Fermi level of the oxide band gap, the study of this issue has remained very limited until now.

In this paper, we focus on the precise analysis of the band structure and the electrical and optical properties of low-temperature-processed SIZO thin films, which contain small amounts of Si from 0.1 to 0.3 wt%. Band gap analysis using a combination of photoelectron spectroscopy (PES) measurements is a very powerful method to investigate the intrinsic properties of semiconductor materials. and revealed its accuracy applied to oxide semiconductor. By varying the levels of Si doping in the IZO TFTs, changes in the TFT electrical performance have been clearly observed, such as the electron carrier concentration, field effect mobility, and threshold voltage. The addition of small amounts of Si to the SIZO thin film demonstrates that the formation of donor-like states can be easily controlled by Si doping^11^. Therefore, it is very important to investigate the relationship between the Fermi energy level and Si doping; Si suppresses the number of oxygen vacancies and hence shifts the Fermi energy level away from the conduction band towards the intrinsic level.

## Results and Discussion

**Figure 1:**
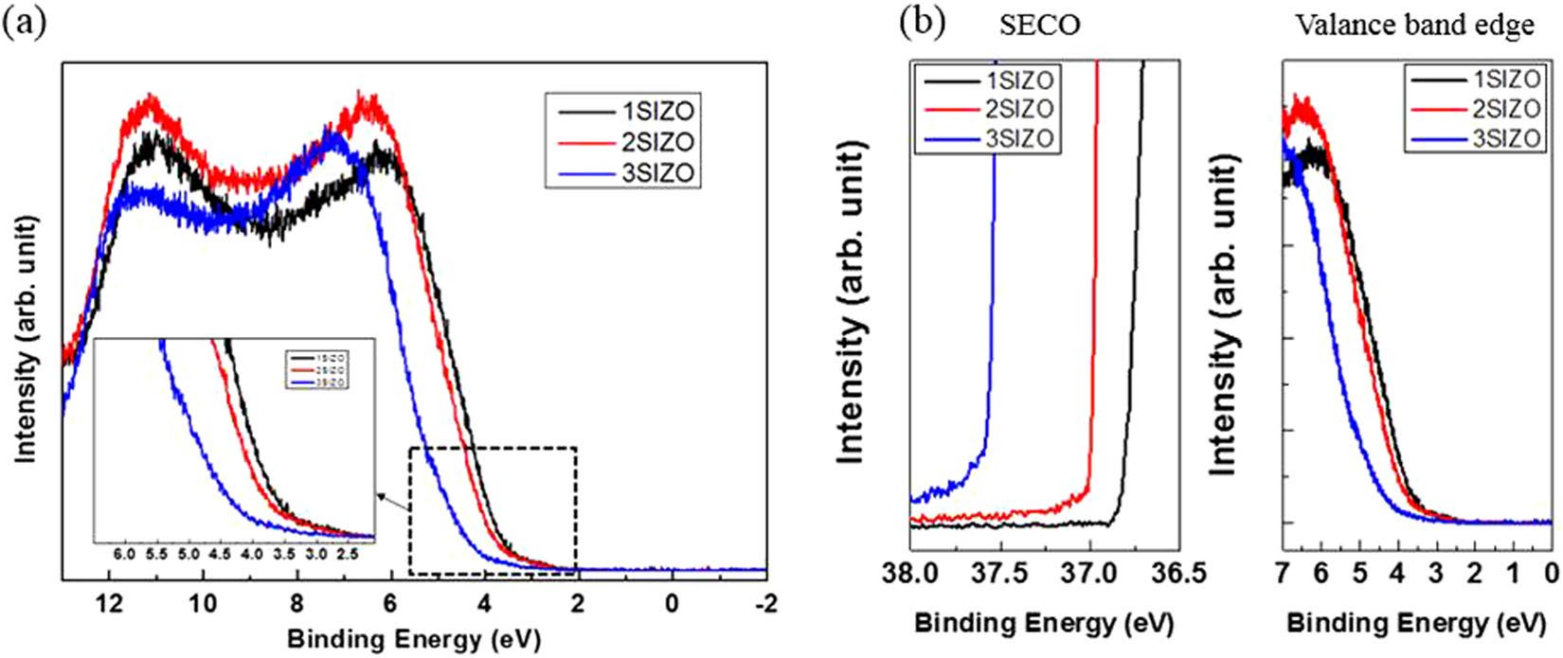
Ultraviolet photoelectron spectroscopy (UPS) analysis of the a-SIZO thin films with different Si contents. (**a**) Valence (E > 0) and conduction (E < 0) band edge spectra measured on 1SIZO, 2SIZO and 3SIZO film using UPS. The band edges onsets are indicated in the inset. (**b**) He II spectra of SECO and valence band edge with varying Si contents.

**Figure 2:**
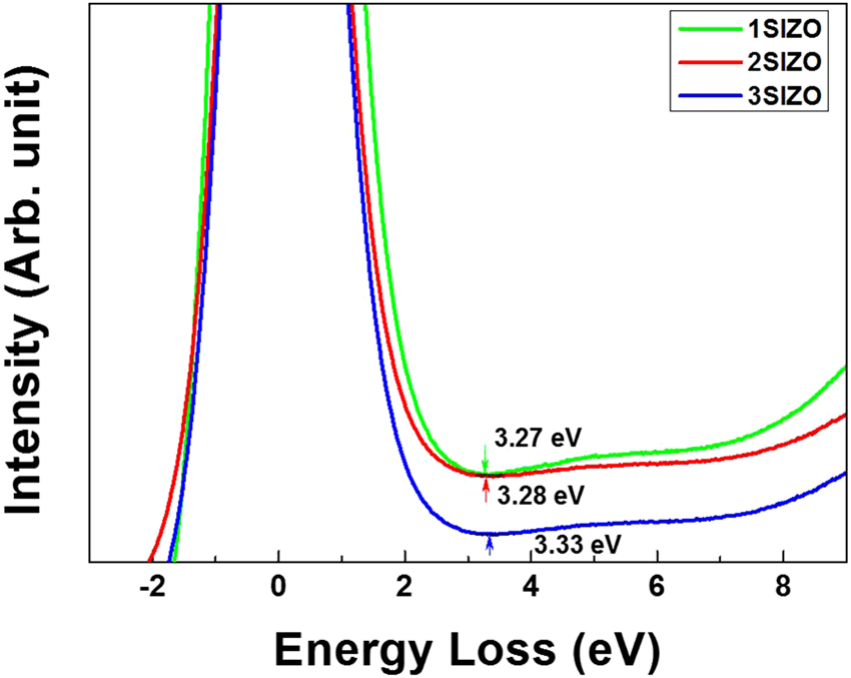
The bandgap measurements of SIZO using Monochromatic He II (hν = 40.813 eV) at Si ratio.

**Figure 3:**
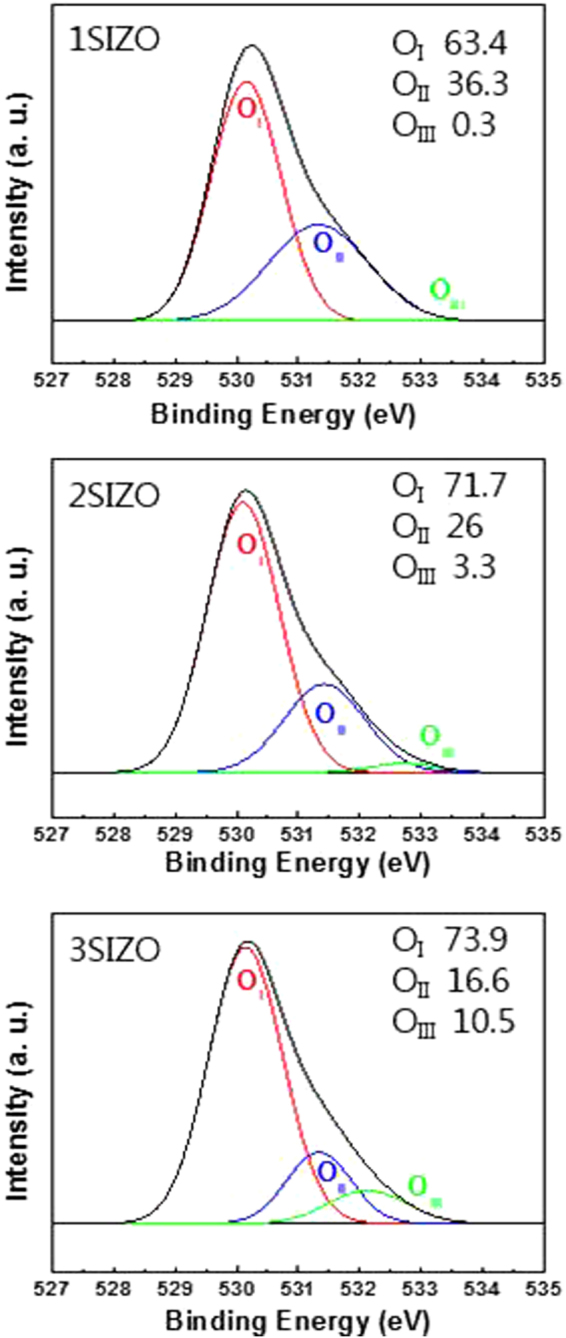
XPS spectra of the O 1 s core level line for the a-SIZO films as a function of Si content.

Figure 1 shows the ultraviolet photoelectron spectroscopy (UPS) results of the SIZO samples with different Si contents. The secondary electron cut-off (SECO) point of each device was determined by fitting a straight line to the low kinetic energy cut-off, and then finding its intersect with the baseline of the spectra^12,13^. In UPS measurements, the exposure of the sample to ultraviolet light causes chemical reactions both the sample surface and any contamination, which usually results in a surface dipole^14,15^. This could cause a significant work function reduction^16,17^. Thus, the minimisation of surface contamination and suppression of UV-induced dipoles are important factors in reliable core level spectroscopy. Hence, electron energy loss spectroscopy (EELS) and Kelvin probe (KP) measurements were conducted before UPS to minimise the formation of UV-induced dipoles. High-quality uniform surfaces were fabricated via the precise control achieved using radio frequency (RF) sputtering in a vacuum chamber. The valence bands of the devices were directly estimated by using the following equation:

1 $${\rm{valence}}\,{\rm{band}}\,({\rm{eV}})=h\nu \,-\,(SECO\,-\,VBM\_{E}_{F})$$

where VBM_*E*_*F*_ is the gap between the valence band maximum (VBM) and Fermi level, and *hν* is the energy of the monochromatic He II emission (40.813 eV). From these UPS results, valence band energies of 7.43, 7.41, and 7.33 eV were obtained for the SIZO films with Si doping of 0.1, 0.2, and 0.3 wt%, respectively. In order to calculate the band gap-alignment energy diagram, EELS measurements were performed. EELS is capable of analyzing electronic and optical properties of oxide materials because the low-energy-loss region reflects the valence and conduction band structures of solids. The band gaps of the SIZO films derived from the EELS measurements were estimated to be 3.27, 3.28, and 3.33 eV with increasing Si content in Fig. 2. By this method, the conduction bands for the films with 0.1, 0.2, and 0.3 wt% Si can be estimated at 4.16, 4.13, and 4.00 eV, respectively. Generally, Si^4+^ is tetravalent and contributes four valence electrons; these may act either as a potential donor or acceptor, called an amphoteric dopant. It has been reported that a low carrier concentration may lead to a low mobility in oxide TFTs due to the suppression and passivation of oxygen vacancies^18,19^. Considering O-bonding, oxygen radicals preferentially react with Si radicals because the most favourable chemical bond-forming reaction in the plasma state gives Si–O_X_. Thus, oxygen radicals react mainly with silicon, prior to either the formation of Zn–O or In–O bonds^20^. The threshold voltage (V_th_) of the 3SIZO TFT was positively shifted by about 7.9 V than these of 1SIZO TFT. This clearly indicates that the electron carrier concentration is decreased by doping with Si, resulting in the positive shift of V_th_ as reported by others^18^ Increasing the Si content also led to a decrease in both the mobility and on/off ratio from 26.6 to 2.7 cm^2^/V s and from 4.2 × 10^7^ to 1.2 × 10^5^, respectively. To understand the origin of the changes in electrical performance, the chemical states of the SIZO (0.1, 0.2, or 0.3 wt% Si) films were examined by X-ray photoelectron spectroscopy (XPS). Figure 3 shows the O 1 s XPS peaks for the SIZO thin films, de-convoluted into three Lorentzian–Gaussian peaks. The dominant peaks located at 530.1 eV (O_I_) are assigned to O_2_^−^ ions binding with the nearest-neighbour metal ions in the lattice; those located at 531.3 eV (O_II_) are associated with the O_2_^−^ ion in oxygen-deficient regions; and those at 532.5 eV (O_III_) are assigned to surface oxygen species, such as hydroxyl groups. The ratios of the peak areas (O_II_/O_total_) were used to estimate the relative quantities of oxygen vacancies. The O_II_/O_total_ ratios were 0.363 for 0.1 wt%, 0.26 for 0.2 wt%, and 0.166 for 0.3 wt% Si. The decrease of this ratio with increasing Si content indicates that Si doping reduces the oxygen vacancy concentration in the films, and that this effect can be clearly characterised by XPS analysis.

**Figure 4:**
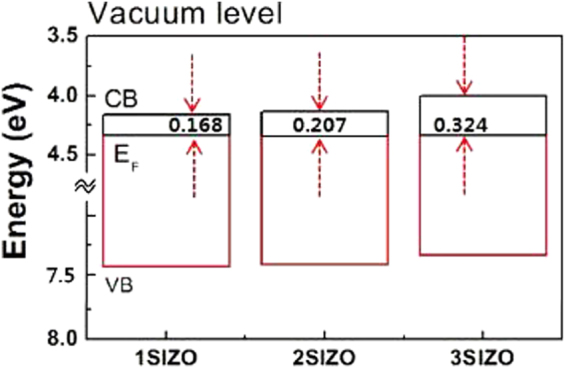
Band structure of the a-SIZO system with varying Si contents. The vacuum level is set to 0 eV for each case.

The electrical properties of the SIZO TFTs confirmed that the field effect mobility (μ_FE_) decreased with increasing Si concentration, indicating that Si was indeed acting as a carrier suppressor through reducing the number of oxygen vacancies in the SIZO system. This is because the M–O bond-strength of silicon (775 kJ/mol) is much higher than those of zinc (385 kJ/mol) and indium (470 kJ/mol)^21^. This result indicates that the oxygen vacancy concentration was decreased by the increase in Si content, resulting to a lower overall carrier concentration. Based on these results, it can be concluded that Si atoms can reduce the number of oxygen vacancies in SIZO films by suppressing vacancy generation. To obtain the absolute work function values, KP measurements were conducted. The measured and calculated energy levels are summarised in Table 1. The gap between the conduction band and Fermi energy, E_c_−E_F_, is the key parameter that expresses the carrier transport and conduction characteristics of the SIZO system. The conduction properties of the SIZO TFTs were found to be strongly dependent on the Si content. As the Si content increased, the gap between the conduction band (E_c_) and Fermi level (E_F_) increased notably. The conduction bands of the films with different work functions were calculated and a schematics energy-level diagram is shown in Fig. 4. Comparing the carrier concentrations, the Fermi level of 1SIZO was located much closer to the conduction band minimum (CBM) than that of 2SIZO and 3SIZO. The difference between the CBM and the Fermi level of the SIZO system became larger as the Si concentration increased. This clearly indicates the decrease of carrier concentration with doping. Weaker bonds require less energy to break and to stimulate an electron to the conduction level. Since Si-O bonding is far stronger than either Zn-O or In-O bonding, charge-carrier generation via formation of oxygen vacancies is more demanding. Therefore, we can control the electrical performance of a SIZO channel device by simply doping with Si.

**Table 1:** Band gap parameters of a-SIZO thin films with different Si compositions.

Sample	Band gap (eV)	E_F_ (eV)	Ev (eV)	Ec (eV)	Ec–E_F_ (eV)
**1SIZO**	3.27	4.33	7.432	4.162	0.168
**2SIZO**	3.28	4.34	7.413	4.133	0.207
**3SIZO**	3.33	4.33	7.336	4.006	0.324

(*x*SIZO denotes *x* = 0.1, 0.2, or 0.3 wt% Si.).

**Figure 5:**
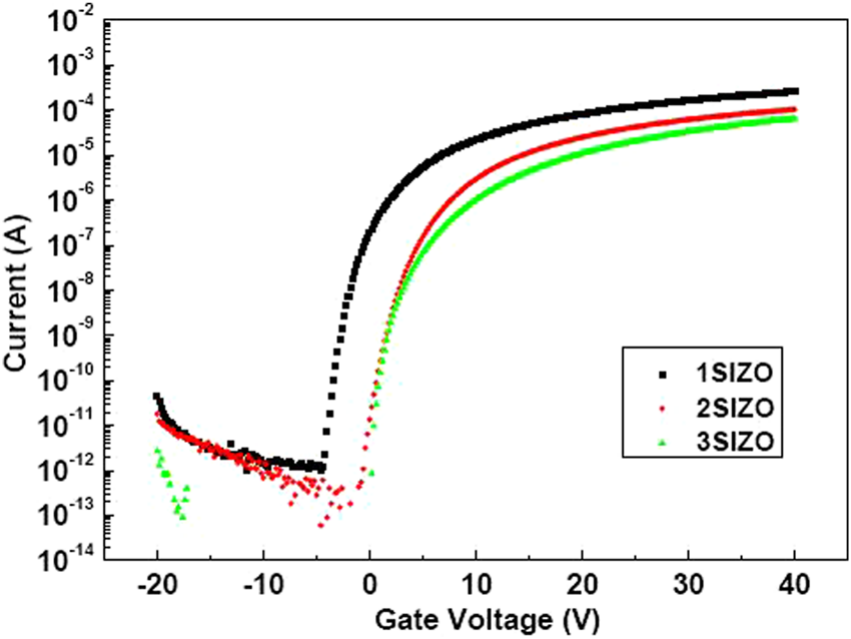
Drain-source current against gate voltage transfer characteristics for the a-SIZO TFTs as a function of Si concentration.

**Figure 6:**
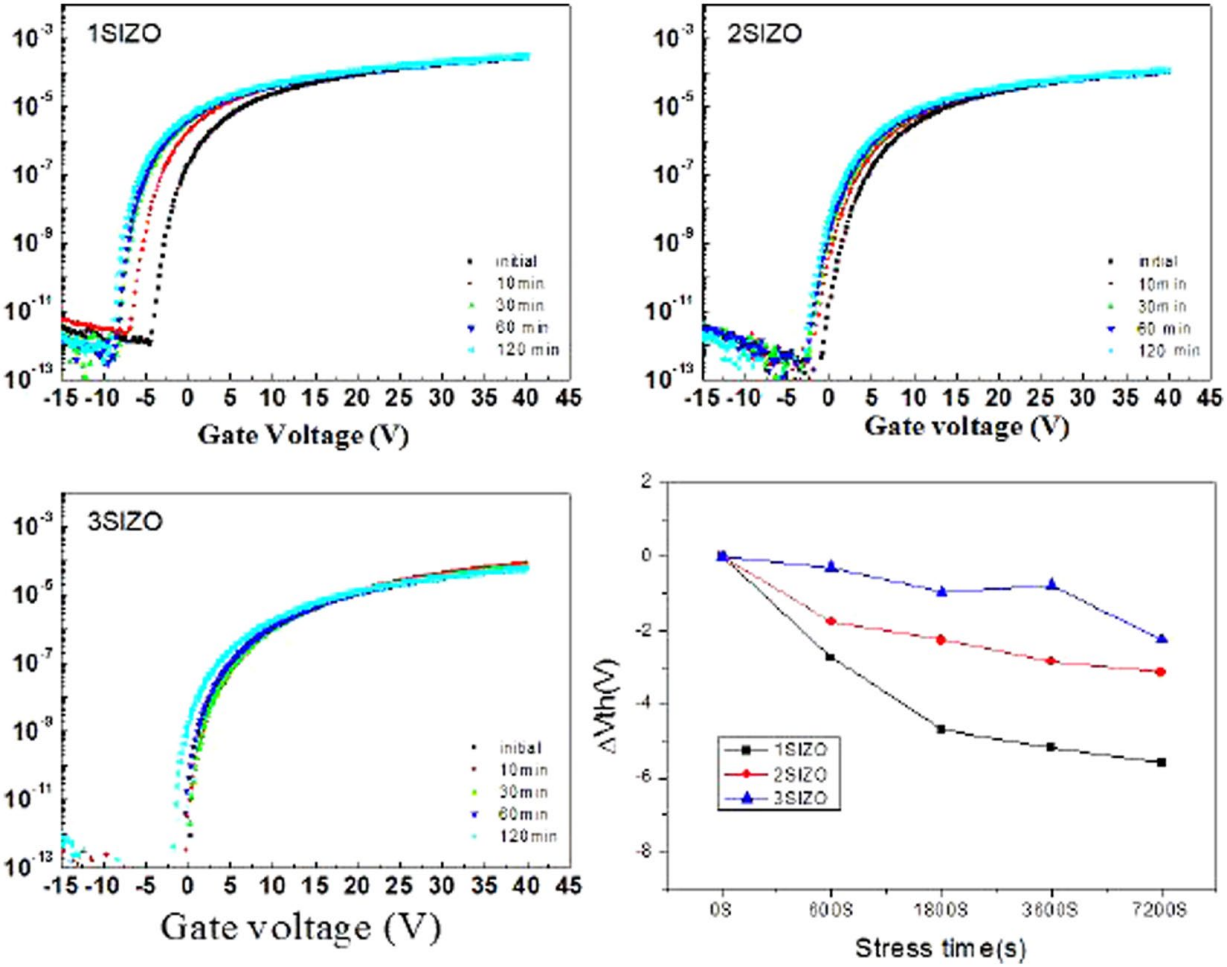
Effect of the NBTS on the transfer characteristics, and the threshold voltage shifts measured at a drain voltage of 10 V, of a-SIZO TFTs with various Si ratios.

**Figure 7:**
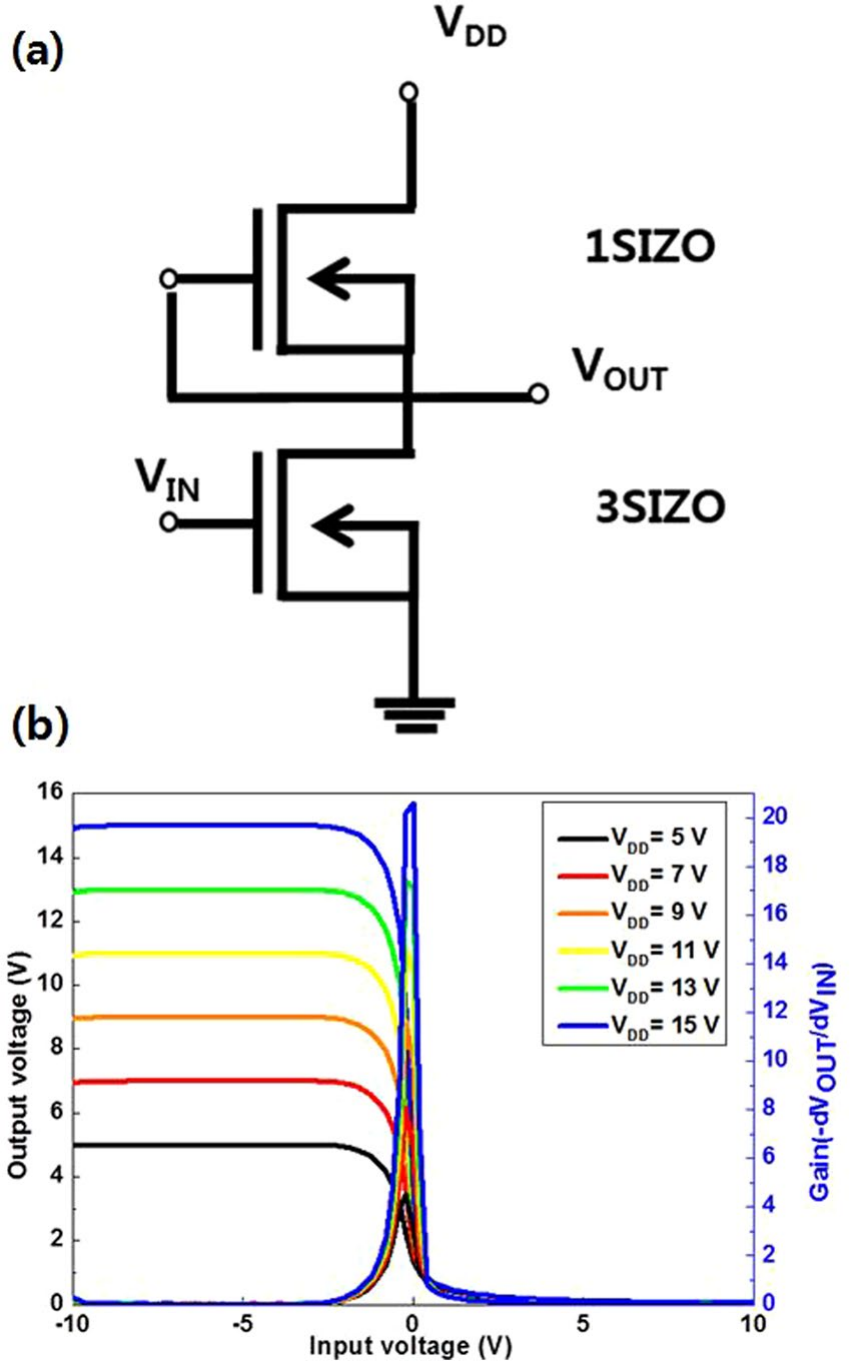
(**a**) Equivalent circuit of the inverter, and (**b**) the voltage transfer characteristic and voltage gain curves of the inverter obtained at various supply V_DD_ values from 5 to 15 V in 2 V steps.

Table 2 summarises the electrical characteristics of SIZO TFTs with various Si concentrations. Figure 5 shows the evolution of the transfer curves obtained at a drain-to-source voltage (V_DS_) of 5.1 V with Si compositions of 0.1, 0.2, and 0.3 wt%. As the Si concentration in the SIZO film increases, the on-current level is lowered and V_th_ is positively shifted. This agrees with the principle that a higher Si content in the SIZO film causes a reduced carrier concentration, since fewer oxygen vacancies are present due to the strong Si–O bond strength^22,23^. Based on this result, it is expected that we can control the electrical properties of TFTs by controlling oxygen vacancies by incorporating Si in the IZO system. Figure 6 shows the change of the SIZO TFT transfer characteristics under a negative bias temperature stress (NBTS) test (gate voltage: V_G_ = −20 V; drain voltage: V_D_ = 0.1 V; and T = 60 °C). When under a negative gate bias, V_th_ shifts negatively, which can be explained by electron charge trapping. The trapping states at the interface between the active layer and gate dielectric can hinder electron movement due to hole trapping^24,25^. The 3SIZO TFT has the lowest ∆V_th_ when compared to other TFTs. The reduction of ∆V_th_ with Si doping means fewer total trap states to capture the electrons at the interface between the active layer and gate dielectric. The electrical properties of the SIZO TFTs confirmed that the field effect mobility decreased with increasing Si concentration, showing that Si was indeed acting as a carrier suppressor through reducing the number of oxygen vacancies in the SIZO system. For large-area applications, the realisation of devices with good electrical properties is essential. Thus, we have fabricated an inverter circuit composed of only n-type oxide TFTs by combining 1SIZO and 3SIZO, as shown in Fig. 7. The electrical properties are summarised in Table 3. These depletion (D)-mode inverter models are operated by the difference in V_th_ that can be simply controlled by changing the Si-doping ratio. These results show that the addition of Si modifies the band gap through tuning the carrier concertation and electrical properties such as field effect mobility and subthreshold swing. High voltage gains of 1SIZO and 3SIZO inverter were obtained, reaching ~20.62 at a supply voltage (V_DD_) of 15 V. The D-mode device a strongly depend on the subthreshold swing (SS)^26^. It is possible to control the transition region and noise margin of the inverter circuits by changing the SS and operating voltage (V_OP_) of the D-mode TFT.

**Table 2:** Electrical parameters of a-SIZO transistors with different Si compositions.

Sample	V_th_ (V)	I_on_ (V)	I_off_ (V)	on/off ratio	Subthreshold swing (V/dec)	mobility (cm^2^/Vs)	S.S (V/decade)
**1SIZO**	0.0	3.15 × 10^−4^	6.2 × 10^−12^	4.2 × 10^7^	0.48	26.6	0.36
**2SIZO**	5.3	2.66 × 10^−5^	1.5 × 10^−10^	2.9 × 10^5^	0.58	5.7	0.30
**3SIZO**	7.9	9.72 × 10^−6^	7.9 × 10^−11^	1.2 × 10^5^	0.36	2.7	0.23

**Table 3:** Voltage gain of 1SIZO and3SIZO-based inverter with different supply V_DD_ values.

V_DD_	5 V	7 V	9 V	11 V	13 V	15 V
Voltage gain	4.53	8.12	11.63	14.65	17.45	20.62

## Conclusion

In summary, high-performance SIZO thin films were fabricated at a low processing temperature of 150 °C, and the effects of Si on the properties of SIZO thin films and SIZO TFTs have been theoretically and experimentally investigated. The relation between the Si content and electrical characteristics of SIZO TFTs were precisely examined, through derivation of the band gaps using a combination of UPS, KP, and EELS measurements. Based on these results, we successfully investigated the crucial role of Si in the electrical performance of SIZO TFTs. As the Si concentration is increased in the intrinsically n-type oxide semiconductor system, the degree of the defect states and mobility decreased, with an accompanying positive V_th_ shift. In the bias stability test, the ΔV_th_ of the SIZO TFTs decreased as Si concentration increased. The incorporation of more Si also improved the V_th_ stability. As the Si content increased, widening of the ΔE_CB_ relative to the vacuum level increases the work function, as was found experimentally in the present work. These changes of the electrical characteristics that are due to the Si concentration affect the band alignments, including the band gap, CBM, VBM, and E_F_. These techniques and the band gap-analysis scheme can be applied to the characterisation of various amorphous oxide thin films, and in the design of high-performance oxide TFTs. Furthermore, it is possible to the annealing temperature by the formation of an oxygen lattice at only 150 °C. Defining the core energy level carrier-transport mechanism of the a-SIZO system with various Si concentrations opens important avenues for the realisation of high-performance a-SIZO-based electrical devices for future applications.

## Methods

SIZO films were deposited on Si wafers terminated with a 200 nm-thick SiO_2_ layer by RF magnetron sputtering at room temperature (RT). This was followed by standard lithography and wet-etching processes to pattern the film.

### a-SIZO film fabrication

The SIZO target was first fabricated using high purity (99.99%) powder mixtures of SiO_2_, In_2_O_3_, and ZnO. Si was incorporated into the IZO (In_2_O_3_:ZnO = 3:1) system at 0.1, 0.2, and 0.3 wt%. The specimens were compressed using a cold isostatic press, then the dried mass was compressed uniaxially at 500 kgf/cm^−2^. The specimens were then sintered at 1250 °C for 3 hr.

### a-SIZO TFT fabrication

To fabricate a-SIZO TFTs with an inverted staggered bottom-gate structure, a-SIZO thin films were deposited on SiO_2_ (200 nm)/p^+^-Si substrates by RF magnetron sputtering. The deposition was performed under a pressure of 0.45 Pa and an RF power density of 0.66 W/cm^2^ at RT in a mixed Ar/O_2_ (95:5) atmosphere. The active thin-film channel layers were 40 ± 2 nm thick. The a-SIZO channel layers were patterned by a wet-etching process, and then annealed at 150 °C for 1 hr in N_2_. Titanium and gold drain electrodes (15 and 50 nm, respectively) were fabricated using a thermal and electron-beam evaporator with conventional photolithography processes. The patterned channels were 250 μm wide and 50 μm long. A passivating poly(methyl methacrylate) (PMMA, A4, MicroChem) layer was spin coated onto the channel layer. All current–voltage (I–V) measurements were conducted using a semiconductor parameter analyser (EL423, ELECS Co., Ltd) at RT. Each experiment was repeated several times.

### a-SIZO film characterisation

The crystallinity of the SIZO films were analysed by X-ray diffraction (XRD, modified PW 1880, Philips) using Cu Kα radiation. Film morphology was observed using a JEOL JSPM-5200 atomic force microscope (AFM). UPS measurements were conducted under ultra-high vacuum (~10^−10^ mbar) with 40.813 eV photon irradiation (He II line). For each sample, the work function was calculated from its UPS spectrum. The difference between the Fermi edge and low kinetic energy cut-off point of the secondary electrons was found from the spectra; the energy of the incident beam was then subtracted. A commercial KP system (KP6500 McAllister Digital Kelvin Probe) was used to measure the work function of the SIZO films. This apparatus measures the contact potential difference (CPD) between a reference plate and the SIZO surface. The probe plate is made of stainless steel with a diameter of 10 mm. During the measurements, the probe plate was electrically connected via a ground to the sample with an accuracy of 2.5 mV. The relative compositions for sputter-deposited a-SIZO thin films were determined from the integrated intensity ratios of Si 2p, Zn 2p_3/2_, In 3d_5/2_, and O 1 s core levels by XPS. To investigate the effects of Si doping in the IZO semiconductor, the energy band diagram of the Si-doped IZO semiconductor was carefully derived by combining the UPS, KP work function, and EELS results.

The SIZO films (Si 0.1, 0.2, and 0.3 wt.%) were profiled with a time-of-flight secondary ion mass spectrometer (TOF-SIMS). These profiles showed that Si, Si–O_2_, In–O, and Zn–O exhibit different distributions inside the film as a function of Si concentration. In-depth analyses using a combination of PES measurements revealed the work function and the band gap shift depended on the Si-doping level in the SIZO films. The band gap is the difference between the CBM and VBM energies in electron volt units. Any change in the band gap value was interpreted as either an energy shift of the CBM or VBM. The band gap analyses were conducted using a combination of UPS (monochromatic He II), ultra-high vacuum KP measurements (KP6500), and EELS (concentric hemispherical analyser (CHA)-type, 23.5 eV energy resolution).
